# A case of meningitis caused by *Ralstonia insidiosa*, a rare opportunistic pathogen

**DOI:** 10.1186/s12879-023-08506-3

**Published:** 2023-08-22

**Authors:** Lindan Liao, Dan Lin, Zhiqiang Liu, Yan Gao, Kezhang Hu

**Affiliations:** Department of Clinical Pharmacy, The First People’s Hospital of Neijiang, 641000 Neijiang, China

**Keywords:** *Ralstonia*, *Ralstonia insidiosa*, Meningitis, Infection

## Abstract

**Background:**

*Ralstonia* is a genus of Gram-negative opportunistic bacteria that can survive in many kinds of solutions and cause a variety of infections. *Ralstonia* spp. have increasingly been isolated and reported to cause infections in recent years, thanks to the development of identification methods such as matrix-assisted laser desorption/ionization time-of-flight mass spectrometry (MALDI-TOF MS) and gene sequencing. However, infections caused by *Ralstonia insidiosa* are still rare. Only a few cases of respiratory infections and bloodstream infections have been reported, none of which involved meningitis. To the best of our knowledge, this is the first reported case of meningitis caused by *R. insidiosa* worldwide. It is necessary to report and review this case.

**Case presentation:**

We report a case of meningitis caused by *R. insidiosa* following lumbar surgery in China. The patient exhibited symptoms of headache, dizziness, and recurrent fever. The fever remained unresolved after empiric antibiotic therapy with intravenous cefotaxime and vancomycin in the initial days. Cerebrospinal fluid (CSF) culture yielded Gram-negative non-fermentative bacteria, which were identified as *R. insidiosa*. As there was a lack of antibiotic susceptibility testing results, clinical pharmacists conducted a literature review to select appropriate antibiotics. The patient’s condition improved after receiving effective treatment with intravenous cefepime and levofloxacin.

**Conclusions:**

Uncommon pathogens, such as *R. insidiosa*, should be considered in postoperative central nervous system (CNS) infections, particularly in cases with unsatisfactory results of empiric anti-infective therapy. This is the first reported case of meningitis caused by *R. insidiosa* worldwide. MALDI-TOF MS provides rapid and accurate identification of this pathogen. The antibiotic susceptibility testing results of *R. indiosa* may be interpreted based on the breakpoints for *Pseudomonas* spp., *Burkholderia cepacia* spp., and *Acinetobacter* spp. Our case presents a potential option for empiric therapy against this pathogen, at least in the local area. This is crucial to minimize the severity and mortality rates associated with meningitis. Standardized antibiotic susceptibility testing and breakpoints for the *Ralstonia* genus should be established in the future as cases accumulate. Cefepime and levofloxacin may be potential antibiotics for infections caused by *R. indiosa*.

## Background

*Ralstonia* is a genus of Gram-negative non-fermentative bacteria. The genus comprises six species. Among them, *Ralstonia mannitolilytica*, *Ralstonia pickettii*, and *Ralstonia insidiosa* have emerged as opportunistic human pathogens in recent years [1]. They caused bloodstream infections [2–4], pulmonary infections [5], septicemia [6, 7], osteomyelitis [8], and CNS infections [9, 10]. Among these cases, sporadic cases and outbreaks of infection were reported. *R. mannitolilytica* and *R. pickettii* are the main components of infectious pathogens, but very few cases related to *R. insidiosa* have been reported. Only a few cases of respiratory and bloodstream infections caused by *R. insidiosa* have been reported worldwide, none of which have involved meningitis [3–6]. Due to the similar characteristics of the genus with other closely related species, identifying the pathogen using traditional methods can be challenging. Meanwhile, there are no standardized antibiotic susceptibility testing methods and breakpoints available for this genus. We present a case of meningitis caused by *R. insidiosa* following lumbar surgery, although the source of the infection is unknown. This is the first reported case of meningitis caused by *R. insidiosa* worldwide.

### Case presentation

A 49-year-old man was admitted to the First People’s Hospital of Neijiang, Neijiang City, China, on May 15th, 2022, due to headache, dizziness, and recurrent fever up to 39℃. A detailed history, clinical examination, and investigations were performed upon admission to our institution. The patient accidentally sustained a fall injury at home two weeks ago. He was admitted to the local hospital and diagnosed with L3 vertebral compression fracture. He underwent lumbar surgery (pedicle screw fixation via a posterior approach) on May 5th, 2022, after which he immediately exhibited symptoms of headache, dizziness, and fever. No blood products were used intraoperatively. Four days before admission to our institution, however, the fever persisted with peaks reaching up to 39℃. Pain and discomfort developed in the lower limb despite receiving anti-infective therapy at the local hospital (details are not available). The patient was then sent to our hospital on May 15th due to his deteriorating condition.

The patient exhibited the following positive signs and symptoms: nuchal rigidity, Kernig sign, Brudzinski sign, Hoffmann sign, and spasticity in the muscles of the upper and lower limbs. The surgical incision healed well without any fluid exudation or local tenderness. Secretions had almost ceased in the drain tubes on both sides.

Laboratory tests revealed an increased white blood cell (WBC) count of 25.32 × 10^9^/L (normal range 3.5–9.5 × 10^9^/L) and an elevated neutrophil ratio (NEUT%) of 93.00% (normal range 40-75%). The erythrocyte sedimentation rate (ESR) was 34 mm/h (normal range 0–15 mm/h). The C-reactive protein (CRP) level was 56.0 mg/L (normal range 0–8 mg/L) and the procalcitonin (PCT) level was 0.14 ng/mL (normal range 0-0.046 ng/mL).

A provisional diagnosis of “meningitis” was made based on the findings mentioned above. Vancomycin (1 g i.v. q12h) and cefotaxime (2 g i.v. q8h) were initiated in anti-meningitic doses to cover the most common bacteria. Blood and CSF samples for bacterial culture should be collected before antibiotic use. Since the patient and his family members initially refused a lumbar puncture, the CSF sample was finally obtained for examination and culture on May 17th (day 3) after detailing the necessity and potential risks involved.

**Fig. 1:**
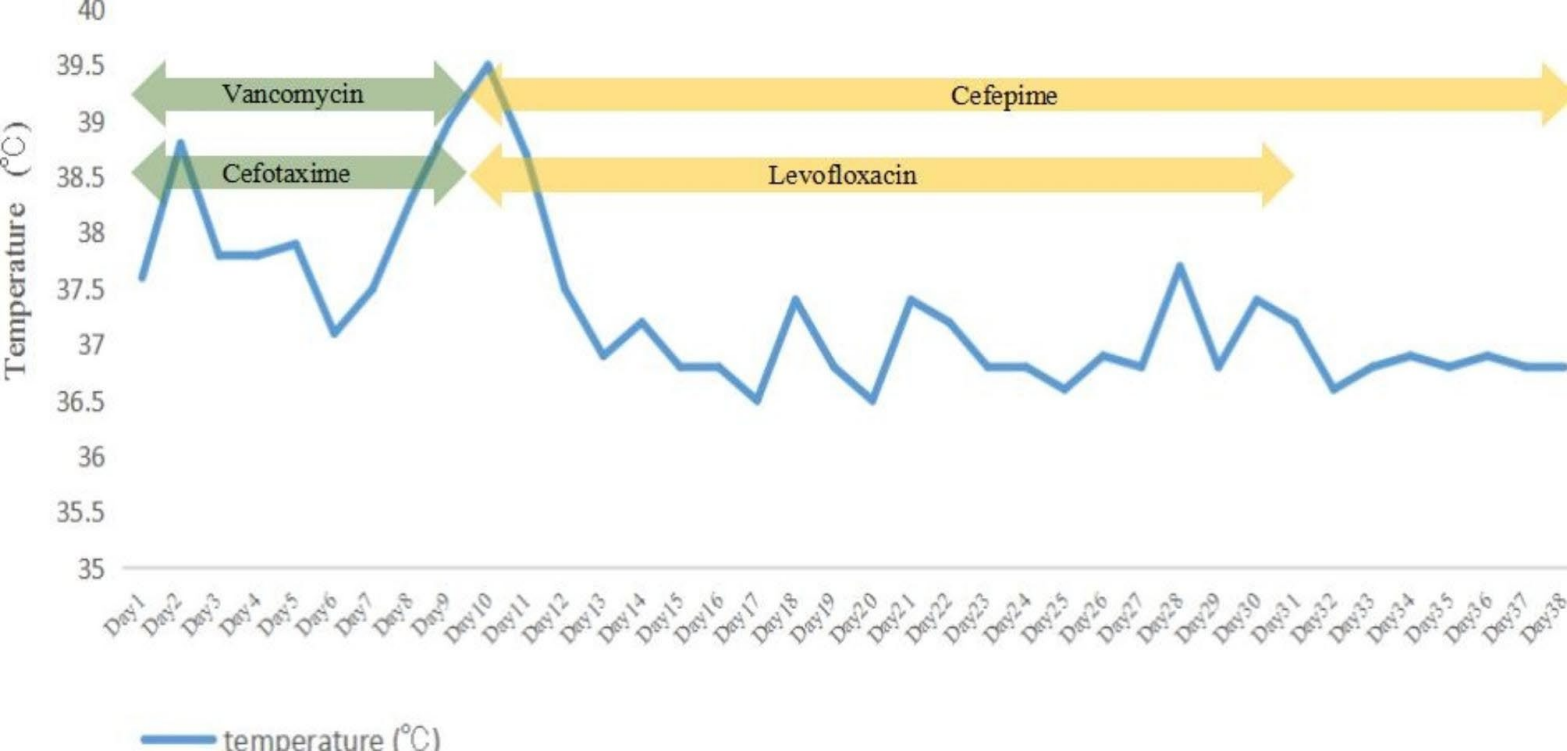
Body temperature and antimicrobial treatment during hospitalization. Vancomycin 1 g i.v. q12h (days 1–8), Cefotaxime 2 g i.v. q8h (days 1–8), Levofloxacin 500 mg i.v. qd (days 9–30), Cefepime 2 g i.v. q8h (days 9–37)

**Fig. 2:**
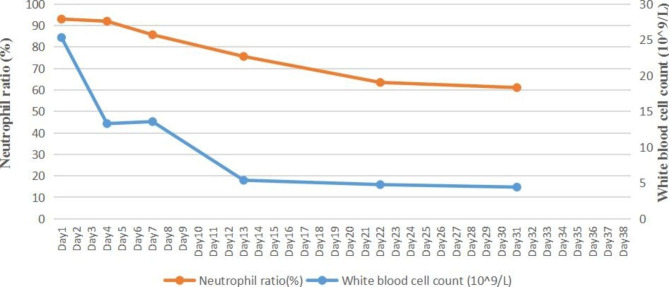
Change in white blood cell count and neutrophil ratio during hospitalization

**Fig. 3:**
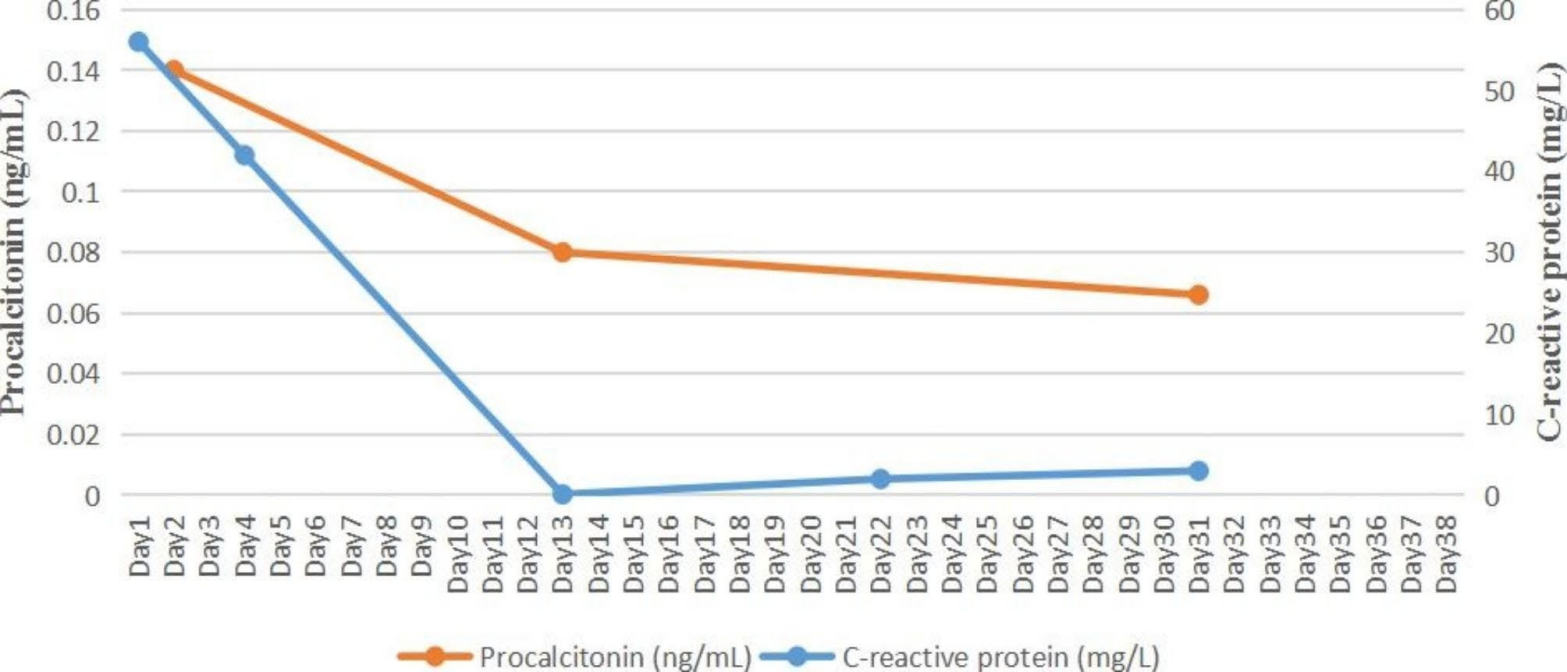
Change in C-reactive protein and procalcitonin during hospitalization

CSF examination showed an elevated protein concentration of 1814.00 mg/L (normal range 150–450 mg/L), a decreased chloride ion level of 117.9 mmol/L (normal range 120.0-130.0 mmol/L), and an increased WBC of 698/µL (20% mononuclear, 80% polymorphonuclear) on May 17th. Generally, normal CSF is cell-free. The normal range of WBC in CSF collected by lumbar puncture is no more than 5/uL, which is composed of lymphocytes and monocytes only in normal circumstances. The patient’s clinical condition improved, including a reduction in headache, normalization of body temperature (Fig. 1), and a decrease in WBC, NEUT%, and CRP on day 4 (Figs. 2 and 3).

However, the recurrent fever persisted, with peaks reaching up to 39.5℃ from day 6 (Fig. 1). CSF culture yielded Gram-negative bacteria on May 23 (day 9) after 6 days of incubation, while the blood culture remained negative. The isolates were automatically identified using the VITEK 2 Compact system with its GN card (BioMérieux, France). They were initially identified as *R. insidiosa* (probability of 98.0%) and later confirmed to be the same pathogen (probability of 99.9%) using MALDI-TOF MS (VITEK MS, BioMérieux, France). *R. insidiosa* was not included in the susceptibility testing database of the VITEK 2 Compact system. Furthermore, there were no standardized antibiotic susceptibility testing or breakpoints available for this pathogen. Therefore, no antibiotic susceptibility results were provided to doctors in the hospital information system. Clinical pharmacists were once again invited to assist with adjusting the medication regimen. As there was a lack of antibiotic susceptibility testing results (which, as mentioned above, were not available in our microbiology laboratory, and this will be explained further in the discussion section), we conducted a literature review on infections caused by *R. insidiosa* to make a treatment decision. The patient was treated with cefepime (2 g i.v. q8h ) and levofloxacin (500 mg i.v. qd ) for 21 days. As the patient’s clinical condition improved, levofloxacin was removed from the subsequent medication regimen considering the potential musculoskeletal adverse drug reaction (ADR) associated with fluoroquinolones. Cefepime (2 g i.v. q8h ) was continued for another 7 days until discharge. The patient’s fever resolved (Fig. 1) with normal WBC, CRP, and PCT levels (Figs. 2 and 3). He was discharged from the hospital on day 38 with negative Kernig sign, Brudzinski sign, Hoffmann sign, and no other complaints. No adverse reactions occurred during the treatment. No recurrence of infection was found within one year of telephone follow-up till we submitted this manuscript.

In this case, possible differential diagnoses of viral meningitis and vertebral osteomyelitis were ruled out. Digital radiography (DR) and magnetic resonance imaging (MRI) of the spinal column showed no evidence of osteomyelitis or acute infection. The increased WBC (698 × 10^6^/L, 20% mononuclear, 80% polymorphonuclear) in CSF is more likely to be seen in cases of bacterial meningitis. This is supported by the positive CSF bacteria cultures.

## Discussion and conclusions

CNS infection typically presents with fever, headache, nuchal rigidity, and altered mental status. Rapid recognition and timely treatment are required due to the high mortality associated with this condition [11, 12]. Meningitis was one of the top 10 causes of disability-adjusted life-years in children under the age of 10 in 2019, according to the Global Burden of Disease Study[13]. For adults, CNS infections often result from otorhinolaryngological procedures, neurosurgical operations, and spinal surgeries. Postoperative CNS infections may manifest as meningitis, encephalopyosis, subdural abscess, and epidural abscess. Among them, meningitis and encephalopyosis are the most common complications in brain surgery [14]. Infections after spinal surgery occasionally involve the CNS. The infection rate varies in different surgical sites and approaches, with lumbar and posterior approaches being more frequently affected than cervical and anterior approaches [15]. In our case, the patient underwent posterior percutaneous pedicle screw fixation in the lumbar, which is more susceptible to infection, as mentioned above.

Coagulase-negative *Staphylococci*, *Staphylococcus aureus*, *Pseudomonas aeruginosa*, *Enterobacteriaceae*, and *Acinetobacter* species are common pathogens in healthcare-associated meningitis [16]. *Ralstonia* spp. have rarely been reported to cause CNS infections. They are environmental pathogens that exist widely in soil and water. The microorganisms can survive under low-nutrient conditions for a long time. They can be found in tap water and various liquid media in hospitals. Since the bacteria can pass through 0.2 μm filters and form biofilms on surfaces, infections caused by *Ralstonia* spp. are mostly attributed to the contaminated medical products or devices, such as hemodialysate, normal saline solutions, blood products, and disinfectants [2, 3, 17, 18].

*Ralstonia* spp. comprises six species. Among them, *Ralstonia pseudosolanacearum*, *Ralstonia solanacearum*, and *Ralstonia syzygii* are considered plant pathogens. However, *R. mannitolilytica*, *R. pickettii*, and *R. insidiosa* have emerged in recent years as opportunistic pathogens causing human infections [1]. *R. pickettii*, previously classified as a member of *Burkholderia* spp., was reclassified as the type species of the new genus *Ralstonia* in 1995. *R. insidiosa*, which was proposed as a new species in 2003, is a bacterium most closely related to *R. pickettii*. Identification key points for the three human pathogenic species in the traditional way are as follows: *R. mannitolilytica* metabolizes only mannitol, *R. insidiosa* metabolizes only nitrate, and *R. pickettii* metabolizes nitrate and arabinose but not mannitol [19]. However, biochemical identification of *Ralstonia* species is a big challenge because the differences between them are not always typical. That can lead to false identification. *Ralstonia* spp. can even be misidentified as other closely related species, such as *Burkholderia cepacia* complex or *Pseudomonas* species, due to similar characteristics. Even the widely accepted commercial biochemical identification systems on the market can sometimes provide ambiguous results. Recent data shows that these pitfalls can be better avoided with the assistance of spectrometry and gene sequencing techniques. New methods, such as MALDI-TOF MS and 16 S rRNA gene sequencing, provide specific species identification. Whole genome sequencing helps to reveal the mechanisms of antibiotic resistance and pathogenicity. MALDI-TOF MS and 16 S rRNA gene sequencing are the most commonly used methods in the literature for accurately identifying the genus *Ralstonia.* Considering the time and cost, MALDI-TOF MS may be a better choice for rapid and accurate identification.

With the development of modern identification methods, there appears to be a notable rise in the incidence of infections caused by *Ralstonia* species, particularly among immunocompromised patients. Nonetheless, these cases were mainly caused by *R. pickettii* and partially by *R. mannitolilytica*. So far, very few cases involving *R. insidiosa* have been reported. Cases associated with CNS infections are extremely rare and have not been reported worldwide. *R. insidiosa* has caused sporadic cases of respiratory infections, bacteremia, neonatal sepsis [5, 6] and outbreaks of bloodstream infections [3, 17, 20]. Two cases of nosocomial bloodstream infections reported in China suggest that *R. insidiosa* may be resistant to conventional antibiotics, including carbapenems. In addition, in that case, the VITEK 2 Compact system (BioMérieux, France) misidentified the pathogen as *R. mannitolilytica* [4].

Since meningitis caused by *R. insidiosa* has not been reported worldwide so far, there is limited information available on anti-infective therapy. It remains a challenge to select the optimal antibiotics for infections caused by *Ralstonia* spp. due to the limited susceptibility data for the pathogen. There are no standardized antibiotic susceptibility testing and breakpoints available for this genus in the European Committee on Antimicrobial Susceptibility Testing (EUCAST) and the Clinical Laboratory Standards Institute (CLSI) standards. In most of the published cases, breakpoints for *Pseudomonas* spp., *Burkholderia cepacia* spp., and *Acinetobacter* spp. are used to interpret antibiotic susceptibility results of *Ralstonia* genus [21]. In fact, the use of CLSI breakpoints for other bacteria to interpret antimicrobial susceptibility results of *Ralstonia* spp. in published case reports is only tentative. Thus, it is not used in our laboratory. That explains why *R. insidiosa* is not included in the susceptibility test database of the VITEK 2 Compact system. Our clinical laboratory chose not to report the susceptibility data for this pathogen. The main limitation of this study is that we did not save the MIC data of the VITEK 2 Compact system for interpretation, and we did not preserve this bacterial strain for retrospective analysis due to limited knowledge of this novel pathogen at that time. This serves as a lesson to us that clinical pharmacists should improve communication with laboratories to obtain more information when encountering uncommon pathogens. We hope that standardized antibiotic susceptibility testing and breakpoints for *R. insidiosa* will be established in CLSI standards as soon as possible.

We conducted a literature review to gather more information on effective therapy for infections caused by *R. insidiosa*, as there is a lack of antibiotic susceptibility data in our laboratory. All the reported cases of infection attributed to *R. insidiosa* are described in Table 1. In these cases, *R. insidiosa* tended to be susceptible to quinolones and sulfonamides but resistant to aminoglycosides. Furthermore, the pathogen was susceptible to β-lactam antimicrobials in most cases. Aminoglycosides, which are essential antibiotics against infections caused by extensively drug-resistant Gram-negative bacteria, appear to be an inappropriate choice for treating *R. insidiosa* infection. That is worth mentioning. In the study of Ryan and Adley [21], *R. insidiosa* showed little difference in antibiotic susceptibility between environmental and clinical isolates. Quinolones and sulfamethoxazole/trimethoprim were the most effective antibiotics against the species. Their findings are consistent with the cases mentioned above. Based on these cases, we chose a treatment regimen for meningitis that includes quinolones and β-lactam antimicrobials. We made this choice based on their susceptibility in most cases, accessibility, mild adverse drug reactions, CSF penetration, and elimination.

**Table 1:** Main characteristics in cases reported of *Ralstonia insidiosa* infections

references	Country	Year	Type of infection	Susceptible	intermediate	Resistance	Antibiotic treatment	Outcome
5,22	Belgium	2005	respiratory infection	Cefotaxime, cefepime, cotrimoxazole, ciprofloxacin, piperacillin/tazobactam, meropenem		Ampicillin, gentamicin, amikacin	Meropenem, cotrimoxazole	discharged
5,22	Belgium	2005	respiratory infection	Cefotaxime, cefepime, cotrimoxazole, ciprofloxacin, piperacillin–tazobactam, meropenem		Ampicillin, gentamicin, amikacin	Piperacillin/tazobactam	unknown
4	China	2019	bloodstream infection	Ciprofloxacin, levofloxacin, ceftriaxone, piperacillin/tazobactam, tigecycline, trimethoprim/sulfamethoxazole, imipenem, meropenem, cefepime, ceftazidime		Amikacin, amoxicillin/clavulanate, ampicillin, aztreonam, gentamicin, polymyxin B, tobramycin, nitrofurantoin	Ceftazidime	discharged
4	China	2019	bloodstream infection	Ciprofloxacin, levofloxacin, ceftriaxone, piperacillin/tazobactam, tigecycline, trimethoprim/sulfamethoxazole	Cefepime, ceftazidime	Amikacin, amoxicillin/clavulanate, ampicillin, aztreonam, gentamicin, polymyxin B, tobramycin, nitrofurantoin, imipenem, meropenem	Ciprofloxacin,cefoperazone/sulbactam	discharged
6	Turkey	2018	Neonatal Sepsis	Ampicillin/sulbactam,,piperacillin/tazobactam, ertapenem, imipenem, trimethoprim/sulfamethoxazole, levofloxacin, meropenem, amikacin, ciprofloxacin, cefotaxime, cefoperazone, sulbactam, ceftazidime, cefepime, eftriaxone, amoxicillin/clavulanate		Ampicillin, gentamicin, cefazolin	Ampicillin/sulbactam, cefotaxime	discharged
17,22	Czech Republic	2011	Bacteraemia	Cefazolin, furantoin		Gentamicin, amikacin	unknown	discharged
20	Philippines	2022	bacteraemia	Cefepime, ciprofloxacin, Imipenem, Meropenem, Trimethoprim/Sulfamethoxazole	Ceftazidime, piperacillin/tazobactam	Amikacin, gentamicin	Cotrimoxazole	discharged
20	Philippines	2022	bacteraemia	Cefepime, ciprofloxacin, imipenem, meropenem, trimethoprim/sulfamethoxazole	Ceftazidime	Amikacin, gentamicin, piperacillin/tazobactam	Cefepime	discharged
20	Philippines	2022	bacteraemia	Amikacin, cefepime, ceftazidime, ciprofloxacin, imipenem, meropenem, trimethoprim/sulfamethoxazole		Gentamicin, piperacillin/tazobactam	Cotrimoxazole	discharged
3	Turkey	2022	bloodstream infection	Levofloxacin, ciprofloxacin		Amikacin, colistin, piperacillin/tazobactam, ceftazidime, tobramycin	adults:ciprofloxacin; minors:unknown	10 adults:5discharged;5 death due to various comorbidities. 3 minors: discharged

The antibiotics selected based on the literature review resulted in a relatively good clinical outcome for this case. In general, however, conducting a literature review is not a commonly recommended approach to guide antibiotic therapy. The general principle of antibiotic use in meningitis should be broad-spectrum empiric antibiotics followed by deescalation, guided by susceptibility patterns of the specifically identified pathogen. This principle should be the standard of care for the majority of infections. As there is a lack of reliable breakpoints for this uncommon pathogen, conducting a literature review may be helpful for institutions with limited resources.

The overall treatment outcome of this case was good. However, there are limitations to our study. As the patient was already infected before this admission, we could not clarify the source of the infection. The patient did not have high-risk factors such as cancer, chronic renal failure, diabetes mellitus, or other immunocompromised conditions described in the literature [22]. As he exhibited symptoms of headache, dizziness, and fever immediately after receiving lumbar surgery, incision infection may be the main cause. However, at that time, environmental samples and related solutions were not preserved for research at the local hospital because the blood and secretion cultures remained negative till the patient was transferred to our institution. It remains unknown whether the infection was a result of contaminated medical solutions or devices. The delay in performing a lumbar puncture is also a limitation that could affect the culture results. Under normal circumstances, CSF is sterile. Considering that *R. insidiosa* was the only bacterium isolated from the CSF and confirmed by MALDI-TOF MS, it was still considered the suspected pathogen in our case. Molecular methods for ruling out other causes of meningitis are currently unavailable in our hospital, which could be a potential area for future research.

In conclusion, this is the first reported case of meningitis caused by *R. insidiosa* worldwide. Uncommon pathogens, such as *R. insidiosa*, should be considered in postoperative CNS infections, particularly in cases with unsatisfactory results of empiric anti-infective therapy. MALDI-TOF MS can provide rapid and accurate identification of this pathogen. The antibiotic susceptibility testing results of *R. indiosa* can be interpreted based on the breakpoints for *Pseudomonas* spp., *Burkholderia cepacia* spp., and *Acinetobacter* spp. Our case presents a potential treatment option for empiric therapy against this pathogen, at least in the local area. This is crucial to minimize the severity and mortality rates associated with meningitis. Standardized antibiotic susceptibility testing and breakpoints for *Ralstonia* spp. should be established in the future as cases accumulate. Cefepime and levofloxacin may be potential antibiotics for infections caused by *R. indiosa*.

## Data Availability

Data sharing is not applicable to this article as no datasets were generated or analyzed during the current study.

## Abbreviations

MALDI-TOF MS: Matrix-assisted laser desorption/ionization time-of-flight mass spectrometry
CSF: Cerebrospinal fluid
CNS: Central nervous system
WBC: White blood cell count
NEUT%: Neutrophil ratio
ESR: Erythrocyte sedimentation rate
CRP: C-reactive protein
PCT: Procalcitonin
DR: Digital radiography
MRI: Magnetic resonance imaging
EUCAST: European Committee on Antimicrobial Susceptibility Testing
CLSI: Clinical and Laboratory Standards Institute
